# Color improvement and stability of orthodontic clear aligners following different cleaning protocols

**DOI:** 10.1007/s00784-026-06861-4

**Published:** 2026-04-10

**Authors:** Aslı Aşık, Yağmur Lena Sezici, Enver Yetkiner, Arzu Aykut Yetkiner, Nazan Ersin

**Affiliations:** 1 Department of Pediatric Dentistry, Faculty of Dentistry, Izmir Tınaztepe University, Izmir, Turkey; 2 Department of Orthodontics, Faculty of Dentistry, Izmir Tınaztepe University, Izmir, Turkey; 3 Department of Pediatric Dentistry, Faculty of Dentistry, Ege University, Izmir, Turkey

**Keywords:** Clear aligners, Color stability, Cleaning agents

## Abstract

**Objectives:**

To evaluate the color improvement and stability of clear aligners after applying different hydrogen peroxide-containing and hydrogen peroxide-free cleaning protocols under simulated in-vitro conditions.

**Materials and methods:**

Clear aligners (Invisalign, Align Technology, San Jose, CA, USA) were immersed in coffee for 7 days (*n* = 24). They were then subjected to cleaning with four different chemical solutions: hydrogen peroxide-free – Group 1: Invisalign Cleaning Crystals (Align Technology, San Jose, CA, USA) and Group 2: Aktident gel (Rego X-ray GmbH, Augsburg, Germany); hydrogen peroxide-containing – Group 3: Corega Proguard (Stafford-Miller Limited, Waterford, Ireland) and Group 4: Steradent Blancheur Pro (Reckitt Benckiser Healthcare Limited, Hull, UK). Specimens were exposed to the different solutions according to manufacturer’s instructions, followed by a second 7-day discoloration cycle. Color (ΔE*, L*, a*, b*) were assessed at baseline, after a 7-day coffee exposure, after cleaning, and after the second discoloration using a spectrophotometer (Vita Easyshade Compact Advance, VITA Zahnfabrik, Bad Säckingen, Germany). Surface alterations were qualitatively analyzed using a scanning electron microscope (SEM).

**Results:**

After the initial discoloration, ΔE values significantly increased in all groups and decreased following cleaning (*p* < 0.01), indicating partial color recovery. However, a subsequent restaining cycle caused a renewed increase in ΔE (*p* < 0.05). Although no significant intergroup differences were detected (*p* > 0.05), a significant intragroup difference between the first and second discoloration cycles was found in the peroxide-containing groups (Corega Proguard and Steradent Blancheur Pro) (*p* < 0.05). A negative correlation between pH and ΔE₂ was observed in the peroxide-free group (*r* = − 0.64, *p* < 0.05).

**Conclusion:**

Although both hydrogen peroxide-containing and peroxide-free cleaning agents effectively reduce discoloration, variations in their pH and chemical composition influenced their efficacy. The differences in surface alterations among the cleaning agents highlight the need for a comprehensive assessment before recommending specific products to patients for aligner maintenance.

**Clinical relevance:**

Clinicians should consider the chemical composition and pH of cleaning agents when advising patients on aligner maintenance to avoid unwanted surface or color alterations.

## Introduction

The use of clear aligners in orthodontic treatment is a relatively new alternative that has gained widespread popularity due to patients’ preference for invisible appliances [1]. While clear aligners are generally considered aesthetically pleasing, there are still concerns regarding their hygiene and maintenance [2].

Clear aligner treatment involves the sequential use of aligners fabricated from transparent materials, each typically worn for one to two weeks [3]. Although designed to resist discoloration during this period, factors such as the consumption of colored beverages, exposure to ultraviolet radiation, and the use of mouthwash are known to compromise their transparency and color stability. Clinicians generally advise patients to remove aligners while eating or drinking to minimize pigment adsorption by the thermoplastic materials. Research indicates, however, that patients frequently disregard these instructions, leading to a reduction in the aligners’ transparency and aesthetic qualities due to the consumption of food and beverages while wearing them [4].

Clear aligners can be fabricated using either conventional thermoforming techniques or directly through three-dimensional (3D) printing. The polymers most commonly used in the production of clear aligners are polyvinyl chloride (PVC), polyurethane (PU), polyethylene terephthalate (PET), and polyethylene terephthalate glycol (PETG). Invisalign (Align Technology, San Jose, CA, USA), contains polyurethane modified with methylene diphenyl diisocyanate and 1.6 hexanediol, are manufactured from a specially engineered thermoplastic polymer material, commercially known as SmartTrack^®^, the precise chemical formulation of which is not publicly disclosed [5, 6]. The polyurethane comprising Invisalign aligners is susceptible to degradation upon extended exposure to elevated temperatures, moisture, and enzymatic activity due to its inherent reactivity. Polyurethane’s rigid segments contribute to its abrasion resistance, while the flexible segments enhance its elasticity. However, its elastomeric composition renders it susceptible to staining from oral exposure to food and beverages. Studies have shown that aligners are particularly stained by beverages such as coffee, tea, and wine [7].

Currently, there is no definitive consensus regarding a specific cleaning protocol to eliminate discoloration on orthodontic clear aligners; however, mechanical or chemical cleaning methods are generally recommended [4]. Commercially available aligner cleaning products are offered in form of tablets, effervescent solutions, or gels, and are designed to inhibit dental biofilm formation, remove discoloration, and prevent halitosis. The hydrogen peroxide in these products is thought to be incorporated by content manufacturers with the purpose of reducing bacterial load on the aligner surface, inhibiting biofilm formation, and eliminating discolorations through oxidative mechanisms. However, the existing literature provides only a limited number of studies evaluating the effectiveness of these products in removing aligner discoloration and their impact on aligner surfaces [2, 3, 8–11]. Moreover, considering that patients continue to wear the same aligners after surface cleaning, the actual influence of such procedures on discoloration remains uncertain.

Color stability is a critical determinant of both the esthetic and functional success of clear aligners, as any discoloration may not only compromise appearance but also signal material degradation through pigment adsorption, component dissolution, or fluid absorption [12–14]. Despite widespread use, the efficacy of cleaning agents in preventing or reversing staining during routine clinical wear remains insufficiently characterized. Previous studies have mainly assessed the immediate cleaning efficacy of these products after staining, without investigating their protective effect against subsequent staining during continued use [2, 7, 10]. Therefore, the purpose of this study was to evaluate the optical properties of clear aligners that were cleaned using four different cleaning agents, hydrogen peroxide–containing and hydrogen peroxide–free, based on the *Commission Internationale de l’Éclairage* (CIE) color scale and color difference parameters by simulating continued intraoral use [15]. The null hypothesis stated that the different cleaning methods would not significantly affect the color stability of clear aligners, either immediately after cleaning or following re-staining.

## Materials and methods

### Sample size calculation and specimen preparation

A priori sample size calculation was performed using the G*Power 3.1.9.4 program (Heinrich Heine, University of Düsseldorf, Germany), resulting in a total of 24 samples (a = 0.05, 1 – β = 0.95, effect size of 0.4) prepared for four independent groups.

Orthodontic clear aligners made of polyester-urethane (Invisalign, Align Technology Inc., San Jose, CA, USA) were cut into segments covering two molar teeth.

### Color measurement protocol

Baseline color measurements were performed using a spectrophotometer (Vita Easy Shade Compact, VITA Zahnfabrik) calibrated according to the manufacturer’s instructions. To ensure consistency, all measurements were conducted under standard D65 lighting conditions against a white background. The spectrophotometer was fixed in a perpendicular position relative to the aligner surface using a prefabricated positioner to maintain consistent geometry and luminosity. Three measurements were taken for each specimen, and the mean* L*, *a*, and b* values were recorded (ΔE_0_).

### Staining and cleaning procedures

All specimens were immersed in coffee for an equal duration of 7 days, after which color was reassessed using the same spectrophotometer (ΔE_1_). Subsequently, the 24 aligners were randomly assigned into the four groups (*n* = 6). Each specimen was placed in 15 mL falcon tube containing 15mL of the respective cleaning solution. For cleaning of the samples, four different cleaning agents, with two hydrogen peroxide-containing and two hydrogen peroxide-free were utilized. The compositions of the cleaning agents are presented in Table 1.

**Table 1:** Composition of the cleaning solutions according to the manufacturers' information

**Group**	**Solution**	**Chemical composition**	**Manufacturer**	**Lot No**
I	Invisalign Cleaning Crystals	Sodium Sulfate, Sodium Carbonate, Sodium Tripolyphosphate, Sodium Dichloroisocyanurate, Sodium Lauryl Sulfate	Align Technology Inc., San Jose, CA, USA	20363B
II	Aktident	Sodium Laureth Sulfate, Aqua, Peg-4, Rapeseed Amide, Glycerol, Mentha Arvernis, Saccharin, Sodium Chloride	Rego X-ray GmbH, Ausburg, Germany	31D2901
III	Corega Proguard	Sodium Bicarbonate, Citric Acid, Potassium Monopersulfate, Sodium Carbonate, Sodium Carbonate Peroxide, TAED, Sodium Benzoate, PEG-180, Sodium Lauryl Sulfate, VP/VA Copolymer, Aroma, Subtilisin, Cellulose Gum, Sodium Nitrite, CI 4209 CI 73015	Stafford-Miller Limited, Waterford, Ireland	SM8P
IV	Steradent Blancheur Pro	Sodium Bicarbonate, Sodium Sulfate, Sodium Carbonate Peroxide, Malic Acid, Potassium Carnate, Sodium Carbonate, Citric Acid, Peg-90, Disodium Lauryl Sulfosuccinate, Aroma, Glucose, Aqua, Sodium Chloride, CI 73015	Reckitt Benckiser Healthcare Limited, Hull, UK	8044602

The cleaning solutions were prepared according to the manufacturers’ instructions:

- Group I (Hydrogen Peroxide–free); one packet of cleaning crystals (Invisalign Cleaning Crystals, Align Technology Inc., San Jose, CA, USA) was dissolved in 90 mL of distilled water and gently shaken for 20 s. Aligners were immersed for 15 min.
- Group II (Hydrogen Peroxide–free); a cleaning solution was prepared by adding 12 drops of cleaning gel (Aktident, Rego X-ray GmbH, Augsburg, Germany) into 90 mL of distilled water and mixed gently. Aligners were soaked for 10 min.
- Group III (Hydrogen Peroxide–containing); one effervescent tablet (Corega Proguard, Stafford-Miller Limited, Waterford, Ireland) was added to 90 mL of distilled water, allowed to dissolve, and gently agitated. Aligners were immersed for 5 min.
- Group IV (Hydrogen Peroxide–containing); one single effervescent tablet (Steradent Blancheur Pro, Reckitt Benckiser Healthcare Limited, Hull, UK) was dissolved in 90 mL of distilled water. Aligners were immersed for 15 min.

Following cleaning, all aligners were rinsed with distilled water, air-dried, and subjected to post-cleaning color measurement (ΔE₂).

### Surface characterization

One specimen from each group was randomly selected for qualitative, illustrative surface evaluation with scanning electron microscopy (SEM) analysis (Apreo S, Thermo Fisher Scientific, USA). Each specimen was labeled to designate material, number, and cleaning method. The specimens were gold-plated, and images were collected at 7.5 kV from 14 mm distance and x10.000 and x 25.000 magnification.

### Color difference calculation and pH measurements

To simulate continued clinical use, all aligners underwent a second 7-day immersion in the coffee solution, after which final color measurements were taken (ΔE₃).

Color was evaluated according to the CIE L*a*b* (CIELAB) color scale under D65 standard illumination. This three-dimensional model defines color using the following coordinates:

- L* represents lightness, measured on a scale from 0 (absolute black) to 100 (pure white).
- a* represents the position on the green–red axis, where negative values indicate a shift toward green and positive values indicate a shift toward red.
- b* represents the position on the blue–yellow axis, where negative values indicate a shift toward blue and positive values indicate a shift toward yellow [15].

Color measurements were repeated three times, and the mean value was calculated. Before taking any measurements, the spectrophotometer was centered on each specimen and calibrated using its own calibration tools. Color assessments were conducted under standard D65 lighting conditions and against a white backdrop by one operator.

- ΔL^∗^ represents the variation between the 𝐿∗ values.
- Δa^∗^ represents the variation between the a∗ values.
- Δb^∗^ represents the variation between the b∗ values.

Color differences (ΔE*) were calculated as: 

$$\varDelta E^*=\sqrt{{(\varDelta\:L^*)}^{2}+{(\varDelta\:a^*)}^{2}+{(\varDelta\:b^*)}^{2}\:}$$

ΔE values were calculated according to time intervals as ΔE_1_ (baseline – after 1 week of discoloration), ΔE_2_ (baseline - after the cleaning procedure), and ΔE_3_ (baseline – after 1 week of discoloration following cleaning). For clinical interpretation, a ΔE value below 1 is generally considered imperceptible to the human eye, values between 1 and 3.3 are considered clinically acceptable, and a ΔE value greater than 3.7 indicates a clinically noticeable and often unacceptable color change [15].

The acidity and basicity of the prepared cleaning solutions were recorded using a pH meter (WTW inoLab pH 720, Xylem –WTW, Germany).

**Fig. 1:**
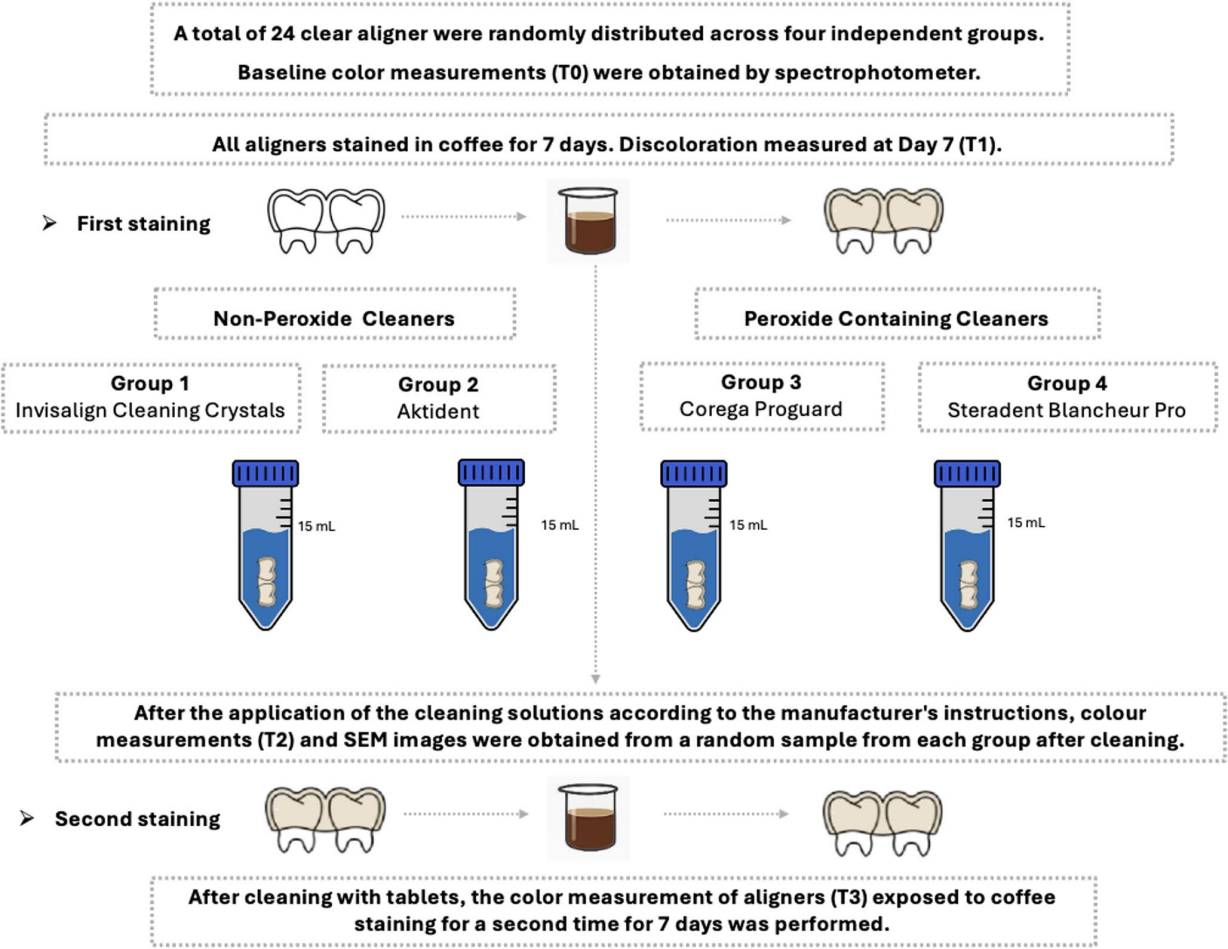
Flow chart of present study

Flow chart of experimental study illustrated in Fig. 1.

### Statistical analysis

Statistical analysis was performed using IBM SPSS Statistics for Windows, Version 27.0 (IBM Corp., Armonk, NY, USA). The normality of data distribution was assessed using the Shapiro–Wilk test.

Descriptive statistics were calculated for the color change across different time intervals. For baseline comparisons of color parameters among the groups were conducted using one-way ANOVA for normally distributed variables (L*and ΔE) and the Kruskal–Wallis test for non-parametric variables (a*, b*).

Intergroup comparisons at each subsequent time point (after staining, after cleaning, and at day 14) were evaluated using repeated-measures ANOVA for normally distributed data or the Friedman test for non-parametric data, followed by Bonferroni-adjusted pairwise comparisons. A paired samples t-test was applied to compare the color change values across baseline-after cleaning and between first-second discoloration.

One-way ANOVA was used to analyze ΔE₁, ΔE_2_, and ΔE₃ across groups. Bonferroni-adjusted post hoc tests were performed to identify pairwise differences.

Pearson correlation coefficients were calculated for normally distributed variables, and Spearman’s rho was applied otherwise, to examine the relationship between solution pH and color change parameters.

The level of statistical significance was set at *p* < 0.05 for all analyses.

## Results

Descriptive statistics of color parameters (L*, a*, b*, and ΔE*) for each group and time point are presented in Table 2. At baseline, no significant differences were found among the groups for any of the color parameters (*p* > 0.05).

**Table 2:** Between-group comparisons of color parameters (L*, a*, b*, ΔE*) at each time point

**Time**	**Factor**	**Group I**				**Group II**				**Group III**				**Group IV**				**p-value**
		**Median±SE**	**Minimum**	**Maximum**	**Mean±SD**	**Median±SE**	**Minimum**	**Maximum**	**Mean±SD**	**Median±SE**	**Minimum**	**Maximum**	**Mean±SD**	**Median±SE**	**Minimum**	**Maximum**	**Mean±SD**	
Baseline	L*	98.00±4.21	73.30	100.00	93.67±10.30	90.75±2.23	83.90	99.20	90.82±5.47	95.30±1.46	90.50	99.70	95.38±3.58	90.65±3.54	76.70	97.70	88.85±8.68	0.455
	a*	−1.20±0.21	−1.60	−0.30	−1.03±0.52	−1.20±0.24	−2.20	−0.50	−1.25±0.59	−1.25±0.18	−1.80	−0.50	−1.15±0.45	−0.70±0.34	−1.50	0.70	−0.62±0.85	0.601
	b*	4.45±0.44	2.70	5.70	4.47±1.09	4.10±0.34	3.00	5.10	4.07±0.84	3.45±0.48	1.70	5.10	3.47±1.18	4.30±0.35	2.30	5.20	4.17±1.10	0.412
	ΔE*	98.13±4.20	73.44	100.04	93.79±10.28	90.86±2.23	84.02	99.30	90.92±5.47	95.43±1.47	90.52	99.78	95.46±3.59	90.77±3.54	76.87	97.83	88.96±8.67	0.457
After discoloration	L*	86.50±2.20	79.80	92.30	86.52±5.38	82.90±1.44	79.60	89.60	83.58±3.55	86.50±2.34	83.20	99.00	88.27±5.74	88.00±3.02	77.20	98.20	87.53±7.39	0.515
	a*	−1.25±0.47	−3.40	−0.30	−1.47±1.17	1.10±0.68	−1.90	2.40	0.75±1.68	−2.25±0.62	−3.10	1.00	−1.72±1.51	−0.35±1.22	−3.20	3.50	0.00±2.99	0.162
	b*	26.40±1.69	19.60	31.20	25.53±4.16	28.00±2.78	22.30	38.20	29.32±6.80	25.90±2.71	19.30	35.10	27.22±6.65	29.95±2.89	18.00	35.80	27.62±7.08	0.708
	ΔE*	90.63±2.26	82.81	95.92	90.30±5.55	89.55±1.10	84.89	92.35	88.83±2.70	90.89±1.92	89.14	101.48	92.64±4.70	91.24±2.30	85.16	100.27	92.16±5.64	0.507
After cleaning	L*	92.85±1.65	87.30	98.50	93.52±4.03	88.20±2.68	80.90	98.90	87.90±6.55	86.90±1.11	82.50	89.70	86.63±2.71	92.50±3.12	77.90	95.50	88.65±7.64	0.191
	a*	−4.10±0.18	−4.40	−3.30	−3.95±0.45	−3.65±0.18	−4.10	−2.80	−3.60±0.45	−3.20±0.34	−3.80	−1.60	−3.05±0.83	−3.60±0.33	−4.20	−2.30	−3.38±0.81	0.135
	b*	19.35±1..88	16.20	28.30	20.82±4.61	20.90±2.16	17.40	32.00	22.10±5.29	20.90±1.91	18.90	31.30	22.60±4.69	19.95±1.82	14.00	27.40	20.45±4.47	0.847
	ΔE*	94.92±2.01	88.85	102.57	95.95±4.92	90.70±2.25	84.62	100.74	90.89±5.51	90.34±1.04	85.44	92.21	89.69±2.56	94.12±2.83	82.17	97.74	91.18±6.95	0.207
After 1 week discoloration	L*	82.90±2.71	73.50	89.80	82.15±6.66	74.95±3.07	67.60	89.10	76.38±7.53	81.35±1.72	79.70	89.20	83.55±4.24	81.95±1.58	76.60	86.50	81.90±3.87	0.182
	a*	−0.25±0.21	−0.60	0.90	−0.10±0.52	0.90±0.22	0.50	1.90	1.08±0.55	1.55±0.36	−0.40	2.00	1.30±0.87	1.05±0.47	−0.40	3.00	1.22±1.17	0.069
	b*	26.80±3.27	17.70	36.60	26.75±8.02	29.25±2.39	22.10	37.10	28.98±5.87	32.85±2.71	18.10	35.80	30.42±6.64	22.80±2.28	17.10	34.00	24.08±5.60	0.424
	ΔE*	87.81±1.83	79.85	91.81	86.82±4.48	81.50±2.64	72.69	91.82	81.96±6.48	88.73±1.35	85.68	94.90	89.17±3.32	85.33±1.07	83.08	89.56	85.58±2.61	0.072

L*: lightness (brightness); a*: red–green axis; b*: yellow–blue axis; ΔE*: total color difference (CIE Lab color space)

ANOVA was applied to normally distributed parameters (L*, ΔE*), and Kruskal–Wallis test was applied to non-normally distributed parameters (a*, b*)

The p-value indicates the difference between groups. Statistical significance was set at p<0.05

After a seven-day immersion in staining agent, the lightness (L* value) decreased in all groups. The comparison of color parameter values from baseline to the first discoloration cycle revealed no significant intergroup differences (*p* = 0.515). Following the cleaning procedures, only the Corega Proguard group exhibited a difference from baseline (*p* = 0.001). After re-staining, the L* values decreased compared to first discoloration in all groups, although the reduction was significant only in the Corega Proguard group (*p* = 0.041).

The a* value presented a slight decrease in all groups after the 7-day discoloration, indicating a minor shift toward the green component. After cleaning, a* values decreased in all groups (*p* < 0.05). Following the re-staining phase, Corega Proguard group demonstrated a significant increase compared to first staining phase in a* (*p* = 0.028).

The b* value increased in all groups after the initial 7-day staining (*p* < 0.05), indicating a marked shift toward the yellow chromatic component. Following cleaning, b* values decreased in all groups (*p* < 0.05), reflecting partial color recovery. After re-staining, no significant changes in b* were observed compared with the first discoloration (*p* > 0.05).

The total color difference from the original baseline (ΔE₁) was first measured after the 7-day staining phase. Following the cleaning phase, the new total color difference from baseline (ΔE₂) was calculated. The ΔE₂ value was lower than the ΔE₁ value in all groups, indicating that cleaning partially reversed the staining and reduced the overall color deviation from the original state. However, in the Corega Proguard group, ΔE₂ was significantly higher than ΔE₁ (*p* = 0.022), meaning cleaning was less effective and the aligners remained more discolored relative to their original color compared to their post-staining state. (Table 3).

**Table 3:** Intragroup comparison of measurements at different time points (L, a, b*, ΔE*)

	**T2/T0**				**T1/T3**			
	**p-value**							
**Group**	L*	a*	b*	**E**	L*	a*	b*	**E**
Invisalign Crystal	0.974	**0.028**	**0.028**	0.641	0.352	0.078	0.600	0.404
Aktident	0.082	**0.027**	**0.027**	0.981	0.127	0.917	0.752	0.110
Corega Proguard	**0.001**	**0.028**	**0.028**	**0.022**	**0.041**	**0.028**	0.345	**0.028**
Steradent Blancheur Pro	0.901	**0.028**	**0.028**	0.257	0.097	0.249	0.345	**0.011**

The p-value was calculated using the paired-samples t-test for normally distributed data and the Wilcoxon signed-rank test for non-parametric data

Bold font highlights significant intragroup differences at p < 0.05

Table 4 summarizes temporal color variations within each group. Time-dependent changes were significant in Invisalign Cleaning Crystals, Aktident gel, and Corega Proguard (*p* < 0.05). In Invisalign Cleaning Crystals, L* decreased from 93.67 ± 10.30 to 82.15 ± 6.66 after the second discoloration (*p* = 0.035), accompanied by a significant change in ΔE* (*p* = 0.002). Similar trends were observed in Aktident gel (*p* = 0.004) and Corega Proguard (*p* = 0.007), while Steradent Blancheur Pro showed non-significant variations in both L* (*p* = 0.079) and ΔE* (*p* = 0.072). Friedman tests further confirmed time-dependent chromatic variations in all groups.

**Table 4:** Within-group time-dependent changes in color parameters (L*, a*, b*, ΔE*)

**Group**	**Factor**	**Test Statistic**	**p-value**
Group I	L*	F(3,15)=8.600	**0.035**
	a*	χ²(3)=13.086	**0.004**
	b*	χ²(3)=13.400	**0.004**
	ΔE*	F(3,15)=10.400	**0.002**
Group II	L*	F(3,15)=13.600	**0.004**
	a*	χ²(3)=14.600	**0.002**
	b*	χ²(3)=14.600	**0.002**
	ΔE*	F(3,15)=9.200	**0.009**
Group III	L*	F(3,15)=12.200	**0.007**
	a*	χ²(3)=15.000	**0.002**
	b*	χ²(3)=14.600	**0.002**
	ΔE*	F(3,15)=8.900	**0.011**
Group IV	L*	F(3,15)=6.800	0.079
	a*	χ²(3)=9.508	**0.023**
	b*	χ²(3)=13.200	**0.004**
	ΔE*	F(3,15)=5.200	0.072

L*: lightness (brightness); a*: red–green coordinate; b*: yellow–blue coordinate; ΔE*: total color difference according to the CIE Lab color system

Friedman test was applied for non-normally distributed parameters (a*, b*), and repeated-measures ANOVA was used for normally distributed parameters (L*, ΔE*)

Statistical significance was set at p<0.05

Intergroup comparison of color change differentials (ΔL, Δa, Δb, ΔE) is shown in Table 5. No differences were observed for ΔE₁ (*p* = 0.202), ΔE₂ (*p* = 0.345), or ΔE₃ (*p* = 0.326) between groups. No differences were found for Δa or Δb values (*p* > 0.05).

**Table 5:** Intergroup comparison of changes across time intervals

**Time Intervals**	**Factor**	**Group I**				**Group II**				**Group III**				Group IV				p-value
		**Median±SE**	**Minimum**	**Maximum**	**Mean±SD**	**Median±SE**	**Minimum**	**Maximum**	**Mean±SD**	**Median±SE**	**Minimum**	**Maximum**	**Mean±SD**	Median±SE	Minimum	Maximum	Mean±SD	
Baseline – after 1 week of discoloration	ΔL₁	−4.10±4.25	−18.80	7.70	−4.37±10.42	−7.20±3.93	−17.80	9.50	−7.20±9.65	−4.20±1.72	−9.80	1.70	−4.72±4.24	−8.80±2.76	−11.70	3.10	−5.63±6.77	0.932
	Δa₁	1.00±0.67	−0.20	4.30	1.37±1.66	−0.40±0.67	−1.30	2.70	0.33±1.65	3.20±0.52	1.00	4.50	3.02±1.28	1.65±0.87	−2.10	3.90	1.22±2.16	0.083
	Δb₁	2.90±2.40	−9.40	5.90	1.22±5.90	−2.90±3.70	−9.90	10.50	−0.33±9.08	1.80±2.08	−1.70	10.30	3.20±5.10	−3.20±4.07	−14.10	7.60	−3.53±9.97	0.509
	ΔE₁	9.49±2.21	5.99	19.58	10.94±5.40	12.79±2.40	4.12	20.84	13.11±5.88	5.64±1.89	4.01	14.47	7.85±4.64	12.94±0.46	11.30	14.58	13.02±1.15	0.202
Baseline - after the cleaning procedure	ΔL2	−1.10±4.33	−12.70	18.90	−0.15±10.62	−3.10±1.34	−8.20	1.20	−2.92±3.28	−9.85±1.35	−11.80	−3.80	−8.75±3.33	−0.20±1.52	−6.10	5.40	−0.20±3.73	0.077
	Δa2	−2.90±0.26	−3.90	−1.90	−2.92±0.66	−2.10±0.32	−3.60	−1.50	−2.35±0.79	−1.70±0.36	−3.30	−0.80	−1.90±0.89	−2.85±0.28	−3.60	−1.80	−2.77±0.71	0.127
	Δb2	13.90±1.91	12.90	24.30	16.35±4.69	16.60±1.96	14.40	26.90	18.03±4.81	16.70±2.25	14.60	29.60	19.13±5.53	16.45±1.60	10.20	22.30	16.28±3.94	0.684
	ΔE2	19.49±1.71	14.42	24.54	19.33±4.19	16.82±2.39	15.07	28.20	18.61±5.09	20.37±1.77	18.09	30.13	21.58±4.36	16.74±1.54	10.81	22.44	16.90±3.79	0.345
Baseline – after 1 week of discoloration following cleaning	ΔL₃	−14.65±6.00	−26.50	14.20	−11.52±14.71	−15.80±2.63	−21.90	−4.00	−14.43±6.45	−11.75±1.48	−17.80	−7.80	−11.83±3.63	−8.05±2.11	−12.90	−0.10	−6.95±5.17	0.519
	Δa₃	1.05±0.32	−0.30	2.00	0.93±0.80	2.10±0.39	1.50	4.10	2.33±0.96	2.50±0.25	1.40	3.10	2.45±0.62	1.95±0.75	−1.10	4.00	1.83±1.84	0.126
	Δb₃	23.20±3.60	12.00	32.20	22.28±8.83	25.05±2.44	18.30	34.10	24.92±5.97	29.40±2.84	13.80	33.30	26.95±6.98	18.50±2.22	13.30	28.90	19.92±5.45	0.344
	ΔE₃	29.53±4.66	13.76	38.91	27.69±11.43	30.68±3.00	19.58	37.53	29.23±7.35	31.83±2.79	16.13	34.51	29.75±6.84	22.26±1.81	15.62	28.91	21.94±4.44	0.326

ΔL* = change in lightness; Δa* = change in red–green axis; Δb* = change in blue–yellow axis; ΔE* = total color difference

The p-value indicates the difference between groups. Significant at p < 0.05

A strong, negative correlation was found between pH and ΔE₂ in Invisalign Cleaning Crystals (*r* = − 0.877, *p* = 0.022), suggesting that lower pH values were associated with greater total color change. No relationships were found for Aktident gel, Corega Proguard, and Steradent Blancheur Pro (*p* > 0.05). Across all groups, ΔE₂ correlated strongly with Δb₂ (*r* > 0.97, *p* < 0.01), confirming that the blue–yellow axis predominantly contributed to the overall perceptible color variation (Table 6).

**Table 6:** Pearson correlation between cleaning solution pH and color change parameters

**Parameter**	**Group I**	**Group II**	**Group III**	**Group IV**
ΔL2	–0.521 (p = 0.289)	0.600 (p = 0.208)	0.506 (p = 0.306)	–0.613 (p = 0.196)
Δa2	0.561 (p = 0.247)	0.277 (p = 0.595)	–0.123 (p = 0.816)	–0.092 (p = 0.862)
Δb2	–0.639 (p = 0.172)	–0.131 (p = 0.804)	0.278 (p = 0.593)	–0.416 (p = 0.412)
ΔE2	–0.877* (p = 0.022)	–0.189 (p = 0.719)	0.204 (p = 0.699)	–0.445 (p = 0.376)

ΔL= change in lightness; Δa= change in red–green axis; Δb= change in blue–yellow axis; ΔE= total color difference; p = significance level; r = Pearson correlation coefficient.*Significant at p < 0.05 (two-tailed)

**Fig. 2:**
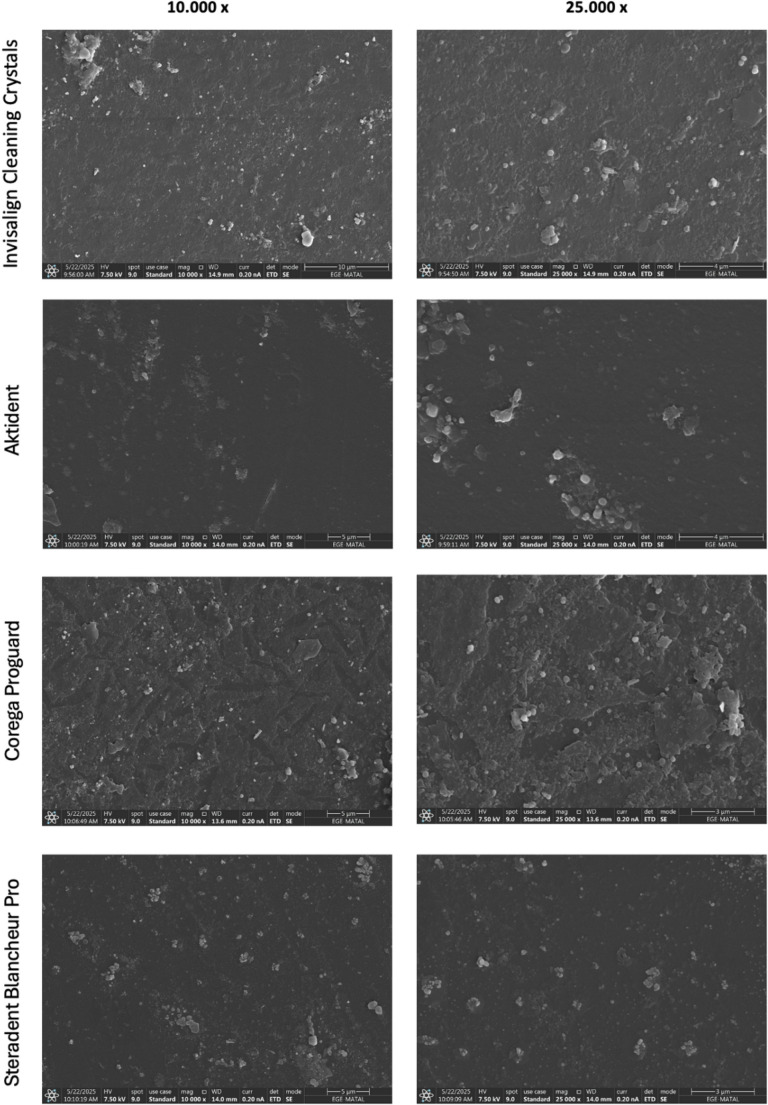
Surface characterization by SEM (10.000x and 25.000x magnification) demonstrating the effects of four different cleaning agents on aligners

Qualitative SEM observation showed at 10.000x and 25.000x magnification (Fig. 2) revealed that all cleaning agents left varying degrees of surface residue. The hydrogen peroxide-based cleaners, Corega Proguard and Steradent Blancheur Pro, resulted in notable residue deposition. Among the non-peroxide cleaners, Invisalign Cleaning Crystals exhibited slightly greater surface residue compared to Aktident gel.

## Discussion

With the increasing popularity of clear aligners, proper maintenance and cleaning remain crucial for preserving esthetics and hygiene. Thermoplastic materials are particularly vulnerable to alterations when exposed to cleansing agents, especially hydrogen peroxide, which may roughen the surface and facilitate stain retention [16]. Although only a limited number of studies have investigated effective cleaning protocols for aligners, most have focused primarily on discoloration outcomes [2, 3, 8, 9]. In contrast, the present study evaluated not only the effects of cleaning after staining but also the impact of cleansing applications over a duration comparable to typical clinical use. The cleaning methods in this study were chosen based on the information gathered from literature. The findings revealed that, the cleaning systems demonstrated comparable color change magnitudes, indicating that none produced superior stain-removal efficacy. However, while no significant intergroup differences were detected, intragroup comparisons showed that cleaning with Corega Proguard resulted in a significantly higher degree of discoloration after re-staining. The hypothesis under investigation was partially rejected in this study that while cleaning agents effectively reduce discoloration, their ability to prevent subsequent pigment adsorption remains limited.

Polyurethanes are extensively used in medical devices and are known to have superb mechanical properties, including abrasion resistance, chemical resistance, ease of processing, and high tensile strength [18]. However, despite their favorable mechanical characteristics, polyurethanes are susceptible to discoloration over time due to light exposure, heat, moisture, and enzymatic activity [19]. These factors can promote hydrolysis and oxidative degradation, ultimately compromising surface integrity during intraoral use. Invisalign aligners are found to be more prone to pigmentation after a 12-hour or a seven-day exposure to coffee [2]. In the present study, increases in b values observed after coffee exposure also indicate a shift toward yellow tones, consistent with chromogenic adsorption on the aligner surface. Such yellowing may also result from mild photo-oxidative changes inherent to polyurethane materials, which tend to form yellow chromophoric structures upon oxidative degradation [3]. The dominant Δb* contribution indicates that discoloration mainly resulted from yellow–blue chromatic shifts caused by adsorption of coffee-derived chromogens.

The ΔE value was used to quantify the degree of color change over the time intervals. For clinical interpretation, previous studies have suggested that a ΔE value greater than 3.7 is perceptible to the human eye, even for untrained observers, and is therefore considered clinically unacceptable for aligners used for aesthetic purposes [15]. Conversely, a ΔE value below 1 is regarded as clinically imperceptible, whereas values ranging between 1 and 3.3 are considered within the clinically acceptable threshold [20]. An increase in ΔE indicated greater color deviation, reflecting a deterioration in color stability [21]. It has been demonstrated that all tested cleaning agents induced time-dependent color changes in aligner materials, consistent with earlier reports on polymeric esthetic appliances exposed to staining agents [2]. Although intergroup differences were not significant, the variations in color stability may be associated with the chemical composition of the cleaning agents. Peroxide-based formulations (Corega Proguard and Steradent Blancheur Pro) contain oxidative compounds such as sodium percarbonate and potassium monopersulfate, which may alter the polymer surface through micro-oxidative degradation and facilitate pigment adsorption during subsequent staining cycles [16]. In contrast, non-peroxide formulations (Invisalign Cleaning Crystals and Aktident gel) rely primarily on surfactant and chelating agents, which effectively remove surface debris without causing notable surface alterations Alweneen et al.., possibly explain their comparatively superior color stability [17]. Corega Proguard includes Tetraacetylethylenediamine (TAED) and potassium monopersulfate, which form a highly reactive oxidative environment and lower the solution’s pH. The strong negative correlation between pH and ΔE₂ in the peroxide-free group suggests that acidic oxidative environments enhance pigment removal but may also induce subtle surface modifications. However, since similar correlations were not detected in other groups, pH alone may not fully explain the variations. Other factors such as surfactant content, effervescence, and exposure time likely play complementary roles. The combination may lead to surface oxidation or micro-etching of the polyurethane aligner, increasing surface roughness and thereby enhancing pigment adsorption during subsequent staining. Accordingly, to evaluate potential surface alterations caused by hydrogen peroxide following the initial staining and cleaning procedures, statistical comparisons were conducted between the first and second discoloration cycles. A difference was found in the Corega and Steradent groups, indicating that hydrogen peroxide-containing cleaning agents may have caused surface or structural modifications affecting subsequent staining susceptibility. This difference may also stem from the specific peroxide concentration, oxidation kinetics, and buffering capacity of the cleaning formulations.

The SEM findings corroborated the optical results. Hydrogen peroxide–free agents (Invisalign Cleaning Crystals, Aktident gel) produced smoother surfaces, while peroxide-containing solutions (Corega Proguard, Steradent Blancheur Pro) left minor surface irregularities and residual deposits. These morphological patterns align with those reported by Lombardo et al., who observed that peroxide-based cleaning methods occasionally caused mild pitting-suggesting effective pigment removal accompanied by limited oxidative micro-damage to the polymer [22]. Levrini et al. [23] found that chemical and mechanical cleaning improved aligner cleanliness without fully eliminating surface residues, while Bernard et al. [2] reported cleaning with either the Invisalign crystals or the Retainer Brite tablets, aligners that had been exposed to tea for 7 days reverted almost back to their initial color. Agarwal et al. [8] also demonstrated decreased translucency of thermoplastic retainers after prolonged exposure to cleaning agents, indicating that optical aging is influenced more by material composition than by the cleaner formulation itself. These findings also agreed with those of studies such as Liu et al. [3] highlighted the combined influence of material type and cleaning protocol on aligner esthetics. However, the present study expanded this scope by incorporating an additional re-staining phase after cleaning and by performing SEM analysis post-cleaning to visualize surface alterations, which was not thoroughly addressed in previous literature. The observed discoloration thresholds in this study generally conformed to those reported Fidan and Gelgor [13].

In the present study, peroxide-containing agents produced more irregular, granular topographies with residual deposits, consistent with incomplete pigment removal and oxidative micro-alteration. Overall, these findings suggest that peroxide-based systems may achieve slightly higher initial cleaning efficacy without statistical superiority, whereas non-peroxide formulations better preserve the polymer surface morphology. Wible et al. is not recommended hydrogen- peroxide as a cleaning solution for polypropylene/ethylene copolymer retainer material due to its powerful oxidizing abilities [9].

Moreover, SEM observations emphasize that the formulation type rather than the mere presence of peroxide appears to be the key determinant of surface alteration. Effervescent tablets, in particular, tended to induce more pronounced topographical changes and residue deposition compared with gel-based formulations [24, 25]. This may be attributed to their mode of action: the rapid release of CO₂ gas creates interfacial turbulence and cavitation, and the collapse of microbubbles likely generates localized mechanical stress that compromises surface integrity and promotes particulate adhesion [26]. Additionally, insoluble excipients (e.g., sodium bicarbonate–citric acid couples, binders, disintegrants) may precipitate and adhere to the microtextured surface after effervescence. In contrast, gel formulations act primarily through sustained chemical dissolution and surfactant mechanisms with minimal mechanical abrasion, thereby preserving aligner surface texture.

Clinically, smoother surfaces reduce pigment re-adsorption and microbial adhesion. Although all agents achieved color recovery within clinically acceptable thresholds (ΔE < 3.7), peroxide-free agents preserve material texture with slightly lower whitening efficiency. Effective patient instructions on cleaning protocols are therefore essential to maintain esthetics and prevent biofilm formation.

Maintaining effective cleaning is essential not only for esthetics but also for minimizing bacterial accumulation on aligner surfaces during prolonged wear [27]. The present findings align with earlier studies emphasizing the role of cleaning procedures in preserving the esthetic properties of clear aligners. Chang et al. [28] demonstrated that both mechanical and chemical methods were effective in limiting discoloration and maintaining translucency in Essix retainers, while Agarwal et al. [8] reported that long-term cleaning practices significantly influenced the color stability and surface integrity of copolyester retainers. In the current study, although all cleaning protocols reduced discoloration, no significant differences were observed between the tested solutions. Since clear aligners are used not only during active orthodontic treatment but also as retainers or during intervals between new aligner sets, the experimental design simulated both short replacement intervals (weekly) and longer periods (biweekly) to reflect clinical reality.

Overall, while all agents effectively reduced discoloration to clinically acceptable levels, none demonstrated superior color-restoring performance. These findings suggest that the optical outcomes of aligner cleaning are more dependent on polymer structure and surface behavior than on the chemical formulation of the cleansing agent. Understanding the optical properties of clear aligners is essential for maintaining esthetic outcomes during orthodontic treatment. Since aligners cover the tooth surface directly, their color and translucency can significantly influence the perceived tooth shade [2]. Any discoloration or surface alteration may compromise esthetic harmony, particularly in the maxillary anterior region where visibility is greatest. Therefore, ensuring the optical stability of aligner materials through appropriate cleaning methods is crucial. Proper cleaning protocols can influence both the esthetic durability of clear aligners and patient satisfaction. Clear instructions from clinicians on correct cleaning methods are essential to support optimal treatment outcomes.

Polyurethane’s resistance to temperature and moisture plays a critical role in its clinical performance [6]. However, repeated exposure to oxidizing agents such as hydrogen peroxide may alter intermolecular interactions within the polymer matrix, potentially affecting its surface integrity and long-term stability [29]. While this study focused on color changes under simulated use, further research should incorporate advanced analytical methods, such as surface roughness and mechanical testing, to better understand how cleaning solutions influence the structural and functional properties of aligner materials. Although an a priori power calculation was performed, the use of a conservative effect size and the resulting sample size (*n* = 6 per group) may limit the ability to detect smaller, yet potentially clinically relevant, differences between cleaning protocols. Future studies with larger sample sizes are warranted to confirm these findings and improve generalizability. Furthermore, the SEM analysis, which was performed on a single specimen per group for illustrative purposes, provides only qualitative insights into surface topography and should not be overgeneralized. Also, further in-vivo studies are needed to establish standardized, evidence-based cleaning recommendations that balance effective decontamination with material preservation.

## Conclusions

After sequential staining, cleaning, and re-staining cycles simulating clinical use, all tested cleaning agents produced partial color recovery in clear aligners but failed to completely prevent subsequent discoloration. No significant differences were observed among the four cleaning systems in terms of overall color change; however, hydrogen peroxide-free formulations better preserved surface integrity. Peroxide containing cleaners exhibited a significant increase in discoloration between the first and second staining cycles, implying that its peroxide-based composition may enhance surface susceptibility to subsequent pigment adsorption. Within the limits of this in vitro study, both peroxide-based and non-peroxide cleaning agents effectively maintained color within clinically acceptable thresholds, suggesting that aligner optical performance depends more on the intrinsic properties of polyurethane than on the specific cleaning formulation used.

## Data Availability

The entirety of the data analyzed in this study has been included in the published article. Upon making a reasonable request, the corresponding author will provide access to the raw data.
